# Supplementary material for: Exploring health literacy pertaining to general wellbeing and chronic disease management among population registered within Primary Healthcare System: A Study protocol
Source: PLoS One. 2025 Oct 7;20(10):e0333194. doi: 10.1371/journal.pone.0333194 (PMC12503302; doi:10.1371/journal.pone.0333194)
Supplement: S1 file — (docx) [file pone.0333194.s001.docx]

**Topic guide for FGD for health care professionals working at PHCC**

**Introduction**

Thank you for participating in this FGD. The purpose of this discussion is to gain insights into the health literacy of service users visiting primary health clinics in Qatar. We aim to understand your perceptions and views and how health literacy can affect the general well-being and the management of chronic diseases of patients and what are the existing mediums utilized fir health literacy at PHCC, current challenges and barriers and recommendations to improve the health literacy levels of population registered at PHCC.

Importance of the Study:

Understanding health literacy is crucial as it can significantly impact health outcomes for service users of primary care services at PHCC. By gathering your experiences and perspectives, we hope to identify areas for improvement in health literacy strategies that can better support general wellbeing of the service users as well as better management of chronic disease conditions.

Confidentiality and Consent:

I want to assure you that your responses will be confidential and will only be used for research purposes. Your personal information will be kept private, and participation in this FGD is entirely voluntary.

Overview of Topics to be Discussed:

During our conversation, we will cover several themes, including your understanding of health literacy, your experiences in managing chronic diseases, preferred health information channels, and your thoughts on existing health literacy strategies provided by primary health clinics.

Duration of the Interview:

The FGD will take approximately 1 hour and 15 minutes.

Opportunity for Questions:

Before we start, do you have any questions about the study or the FGD process?

**Broad Theme 1: Understanding Health Literacy**

Sub-Theme: Definitions and Perceptions

- How would you define health literacy in your own words?
- Can you share any experiences that shaped your understanding of health information?

Sub-Theme: Importance of Health Literacy

- In what ways do you believe health literacy affects your general well-being of service users accessing primary care services at PHCC?
- How do you think health literacy impacts the management of chronic conditions?

**Broad Theme 2: Health Management and Chronic Diseases**

Sub-Theme: Experience of Managing Chronic Diseases

- Can you describe your experience with managing patients with chronic conditions?
- What role does health information play in management such patients?

Sub-Theme: Barriers to Effective Management

- What challenges do you feel the services users might face in understanding health information regarding your condition?
- Are there any specific aspects of chronic condition that service users might find difficult to manage due to a lack of information?

**Broad Theme 3: Preferred Health Literacy Channels**

Sub-Theme: Access to Health Information

- What sources of health information are you aware of that are currently used by service users (e.g., healthcare providers, the internet, family, and friends)?
- In your opinion how effective are these sources in providing service users with reliable health information?

Sub-Theme: Channel Preferences

- What formats of health information do you prefer (e.g., printed materials, online articles, videos, face-to-face consultations)?
- In your opinion what would the service users prefer (the various channels of health literacy) to receive important updates about their health condition?

**Broad Theme 4: Evaluation of Existing Health Literacy Strategies**

Sub-Theme: Awareness of Available Resources

- Are you aware of any health literacy programs or resources offered by the Primary Health Care Corporation?
- How did you learn about these resources, and do you find them helpful?

Sub-Theme: Gaps in Current Strategies

- What improvements do you think can be made to existing health literacy strategies in your primary health clinic?
- Are there specific topics or areas where you feel additional information is needed?

**Broad Theme 5: Engagement and Support**

Sub-Theme: Support from Healthcare Providers

- In your experience, what is the role of healthcare providers like yourself to support the understanding health condition of service users accessing primary care services at PHCC?

Sub-Theme: Community and Peer Support

- Do you believe peer support can enhance the understanding of health information of service users? If yes, how?
- Are there community resources you think could help improve health literacy among patients with chronic diseases?

**Conclusion**

- We covered important themes in this FGD including your understanding of the concept of health literacy, your experiences managing chronic diseases, preferred health information channels, and your thoughts on existing health literacy strategies provided by primary health clinics.
- Is there anything that you would like to add which wasn’t discussed or covered in the FGD.
- Thank you for your participation and sharing your invaluable insight.
