# Supplementary material for: Exploring health literacy pertaining to general wellbeing and chronic disease management among population registered within Primary Healthcare System: A Study protocol
Source: PLoS One. 2025 Oct 7;20(10):e0333194. doi: 10.1371/journal.pone.0333194 (PMC12503302; doi:10.1371/journal.pone.0333194)
Supplement: Attachment — Submitted filename: Reply to review comments.docx [file pone.0333194.s002.docx]

1. It appears that you did not upload a Track Changes version of your manuscript file, which highlights any changes you made to your previous submission PONE-D-24-35863.

Authors must submit Track Changes MS files as required by the journal on new submissions of previous PLOS ONE MSs. At this time, please upload a Track Changes version of your manuscript to your File Inventory as an ‘Other’ file. Please feel free to reach out to us at [plosone@plos.org](mailto:plosone@plos.org) if you have any questions.

**Response: Thank you. The track change version has the ethics approval clearly mentioned with the reference number and now attached as an ‘Other’ file.**

2. We note you did not include a copy of the original, (signed or stamped) dated IRB/research ethics committee approval letter or waiver for all study sites as an ‘Other’ file. In keeping with our submission guidelines, please upload the approval letter or waiver as an ‘Other’ file. More information related to the relevant journal policies is available from: [https://journals.plos.org/plosone/s/submission-guidelines#loc-guidelines-for-specific-study-types]

**Response: Thank you for highlighting this. We have now attached the IRB approval letter.**

3. Please note that the Study Protocol article type is only suitable for proposals of studies that have not yet started or that are ongoing. Studies that have completed either recruitment or data collection will not be considered. For further information, please see <https://journals.plos.org/plosone/s/submission-guidelines#loc-study-protocols>. Please ensure that your Methods section reports the status and timeline of the study, including an estimate of when A) participant recruitment will be completed (if applicable), B) data collection will be completed and C) results are expected. If any of these stages have already been completed, this should be indicated in the Methods section.

**Response: The timelines are now specified in the methods section as kindly suggested.**
