# Supplementary material for: Exploring health literacy pertaining to general wellbeing and chronic disease management among population registered within Primary Healthcare System: A Study protocol
Source: PLoS One. 2025 Oct 7;20(10):e0333194. doi: 10.1371/journal.pone.0333194 (PMC12503302; doi:10.1371/journal.pone.0333194)
Supplement: Attachment — Submitted filename: Review feedback-PONE-D-24-46029.docx [file pone.0333194.s003.docx]

**Review feedback: "Exploring health literacy pertaining to general wellbeing and chronic disease management among population registered within Primary Healthcare System: A Study Protocol"** (Manuscript No.: PONE-D-24-46029)

This protocol addresses a timely and highly relevant public health concern—health literacy in chronic disease prevention and management within the primary care setting. The study’s design, ethics compliance, and clarity of objectives demonstrate strong potential to contribute valuable insights to health systems in Qatar and similar contexts. The submission is generally well-prepared for publication, pending minor clarifications.

**Strengths**

1. **Relevance and Timeliness**
   The focus on health literacy in the context of chronic disease management directly aligns with national and global priorities, particularly WHO health promotion strategies.
2. **Rigorous Sampling Strategy**
   The use of stratified random sampling from PHCC’s registry enhances representativeness across age, gender, and nationality, improving generalizability.
3. **Validated Instrument**
   Use of the HLQ in both English and Arabic strengthens the reliability and cultural appropriateness of the tool.
4. **Mixed Methods Approach**
   The integration of both quantitative and qualitative phases will provide a richer understanding of the issue under study.
5. **Ethics and Transparency**
   IRB approval, consent processes, and attached documentation meet international ethical standards.
6. **Document Completion**
   Required materials, including tools, translated forms, and supplementary documents, have been submitted.

**Suggestions for Improvement**

**1. Missing Demographic Data in the Study Tool**

The submitted Health Literacy Questionnaire (HLQ) appears to lack demographic questions (e.g., age, gender, education level, nationality). This is a critical omission for the following reasons:

- **Descriptive analysis** requires participant characteristics to interpret findings meaningfully.
- **Subgroup analysis** is necessary to identify disparities or variations in health literacy.
- **Equity considerations** – health literacy is often shaped by socioeconomic and cultural factors; excluding demographics limits the ability to draw meaningful conclusions and policy recommendations.

**Recommendation**:
Clarify whether demographic data will be obtained separately (e.g., through PHCC registry) or amend the questionnaire to include a brief demographic section. If already planned, this should be stated clearly in the methods section.

**2. Participant Recruitment Details**

- The assumed 2% response rate should be supported with rationale or precedent from similar PHCC-based studies or surveys in Qatar. This adds credibility to the feasibility of the recruitment plan.

**3. Qualitative Sampling**

- While non-probability sampling is acceptable in qualitative research, it would be beneficial to mention efforts to ensure diversity (gender, education, cultural background) in interview participants to reduce potential bias.

**4. Data Analysis Plan**

- Although HLQ scoring methods are referenced, the manuscript would benefit from a concise overview of intended statistical methods, including any subgroup analyses, correlation assessments, or use of regression models.

**5. Conceptual Framework (Figure 1)**

- The figure is valuable but visually complex. A clearer flow with consistent formatting and labeled sections would improve readability for international readers unfamiliar with Qatar's healthcare system.

**6. Language and Style**

- Minor grammatical and structural edits are recommended. For example:
  - Original: “Improved compliance with treatment leading to effective management…” Suggested: “…which may improve treatment compliance and disease management.”
- **Data Availability Statement**: While the current version is compliant, consider stating your intention to deposit anonymized data post-study (e.g., in a public repository or institutional archive).

**Recommendation: Minor Revisions**

The study protocol is methodologically sound, ethically appropriate, and well-aligned with current health system needs. Minor adjustments related to demographic data collection, recruitment rationale, and clarity of analysis plan are needed. Once addressed, this work will make a meaningful contribution to the literature on health literacy and chronic disease management in the region.
