# Supplementary material for: Exploring health literacy pertaining to general wellbeing and chronic disease management among population registered within Primary Healthcare System: A Study protocol
Source: PLoS One. 2025 Oct 7;20(10):e0333194. doi: 10.1371/journal.pone.0333194 (PMC12503302; doi:10.1371/journal.pone.0333194)
Supplement: Attachment — Submitted filename: Revision review feedback-PONE-D-24-46029.docx [file pone.0333194.s005.docx]

**Revision review feedback**

The revised manuscript demonstrates significant improvement over the initial version. It now includes expanded methodological clarity, better integration of supporting literature, ethical considerations, and enhanced structure, making it a promising contribution to the field of public health in Qatar and beyond. However, several **minor but important improvements** are still required for optimal clarity and quality.

**Methodology Enhancements**

- Ensure **consistency of terminology**: For example, “non-probability convenience sampling” and “purposive selection” are both mentioned—be clear on how diversity is achieved in each phase.
- **Timelines** are helpful but could benefit from more detail regarding **data analysis** phases.

**Quantitative Analysis – Clarified but Could Improve Further**

- A concise overview of **planned statistical analyses** is still needed.
  - A subgroup analyses using ANOVA or t-tests and assesses associations using regression models to evaluate factors associated with health literacy scores.
  - Consider mentioning whether weighting adjustments will be used to compensate for the stratified sample.

**Qualitative Approach – Improved with FGDs**

- The inclusion of themes in the topic guides is useful. Consider condensing the **description of FGD logistics** (e.g., room arrangements, consent) for brevity unless required by journal format.

**Patient and Public Involvement (PPI)**

- PPI is acknowledged, and a section is now added.
- However, more **reflective depth** is needed. Consider stating how PPI will be integrated in future research phases.
