# Supplementary material for: Exploring health literacy pertaining to general wellbeing and chronic disease management among population registered within Primary Healthcare System: A Study protocol
Source: PLoS One. 2025 Oct 7;20(10):e0333194. doi: 10.1371/journal.pone.0333194 (PMC12503302; doi:10.1371/journal.pone.0333194)
Supplement: Attachment — Submitted filename: response_to_reviewer_comments_auresp_3.docx [file pone.0333194.s007.docx]

**Response to reviewer comments**

**Reviewer #3: The study addresses an important and under-explored aspect of public health, particularly in multi-ethnic populations and health systems facing rising rates of chronic disease.

Strengths of the Manuscript:

The rationale for the study is well established, highlighting the links between health literacy, healthcare access, compliance, and chronic disease outcomes. The research objectives are clearly stated and justified.

The use of a robust mixed-methods design, combining quantitative (HLQ survey) and qualitative (service user interviews) approaches, is appropriate for capturing both the breadth and depth of health literacy issues in this population.

The methodology is described in sufficient detail to allow for replication. The stratified random sampling, use of a validated tool in both English and Arabic, and consideration of non-response rates are notable strengths.

The protocol is generally well written, presented in clear and standard English, and is logically structured. Ethical approval has been obtained, and plans for data sharing comply with PLOS ONE requirements.**

**Response: Thank you for the positive feedback and your kind review (of the two previous occasions review 1 and 2) which has strengthened the study protocol scientifically. We are truly grateful and have carefully considered the points highlighted in your 3^rd^ review and have highlighted all the points which were suggested. Thank you for your time and continued support.**

**We have elaborated on the suggested details and signposted for your kind review. Each comment is responded separately, and we have mentioned in which section of the protocol the comment is addressed in the track changes document (main manuscript).**

**Points Requiring Attention and Suggestions:

Response Rate and Non-Response Bias:
The protocol anticipates a very low survey response rate (2%) and addresses this with oversampling. While this is pragmatic, the potential for non-response bias remains. Please consider describing additional strategies to encourage survey participation and discussing how non-response bias will be assessed or mitigated in the analysis.**

**Response: Thank you for the important point. We have highlighted in the protocol that the registered users will be sent reminder SMS after two weeks to address potential non-response bias. Moreover, as kindly suggested, we have further described in the analysis component employing appropriate statistical techniques to address the non-response bias and amend expected deviations in the population representativeness by the actual sample.  This is highlighted under the data analysis plan for HLQ questionnaire component of the protocol as shown below:**

**‘The sampling method used will enable valid subgroup analysis by ensuring an adequate sample size in each subgroup. However, any calculated summary measure for the population parameter will be biased by deviations from the sampling frame. This type of bias is addressed by using appropriate subgroup weights, Table 1. Sensitivity analysis (a type of quantitative bias analysis) may be considered to measure the magnitude of sample deviation from the planned sample’.**

**Qualitative Sampling and Diversity:
The qualitative arm relies on convenience sampling, which could limit the diversity of perspectives. Please clarify any steps that will be taken to ensure heterogeneity among interviewees (e.g., sampling across different clinics, demographic quotas) and describe how data saturation will be monitored and determined.**

**Response: Thank you for the important point. We have highlighted in the last paragraph of the discussion component (track changes and clean version of the manuscript):**

**‘Another limitation could be the non-probability convenience sampling strategy to recruit service users to conduct interviews to capture their perceptions regarding the different health literacy channels. To address this issue, we will conduct interviews in different primary care clinics (out of the 32 clinics spread across the state of Qatar) till saturation of data is achieved** **(a point where no new concepts, ideas or themes are emerging, a concept which is evidence based and we have provided the reference) and will aim to include equal representation of male and female participants considering their ethnic backgrounds. Moreover, the FGD will include a diverse cohort (multidisciplinary & multiethnic) of health care providers (HCPs) involved in providing primary care in PHCC** **which will establish that the findings are generalizable, and the sample is representative’.**

**Integration of Mixed-Methods Findings:
Although both quantitative and qualitative methods are detailed, the protocol would be strengthened by a brief description of how these datasets will be integrated during analysis and interpretation to provide a comprehensive understanding of health literacy in the target population.**

**Response: Thank you for the important point. The immediate and the long-term outcomes of the research (qualitative and quantitative findings) is depicted and outlined in figure 2 as kindly highlighted in your comment. As suggested, we have highlighted in the manuscript (first paragraph of discussion, neat and track changes version):**

**‘The mixed methods study aims to assess the existing health literacy channels operating within the primary care health system and to identify the existing gaps and challenges from key stakeholders’ perspectives including both the services users registered with PHCC and the health care professionals. The key objective of the study also includes determining the health literacy levels of service users at population level. The findings of the HLQ highlight health literacy levels at population level and its interrelationship with the sociodemographic indicators whereas the in-depth qualitative investigation highlights the various factors nested within the SEM in context to health literacy of service users accessing primary care services and identifying the potential gaps and challenges. These findings will be triangulated to design an evidence-based health literacy framework which can be utilized for service design and re-design to deliver optimal patient centered primary health care services within the country & modelled in similar health care settings geographically’.**

**Operational Details for Replicability:
While the methodology is overall well-described, providing additional information on interviewer training, data management and security (especially for online data and interview recordings), and specific qualitative analysis procedures (e.g., coding framework, thematic analysis steps) would enhance transparency and replicability.**

**Response: Thank you for the important point. The qualitative component will be carried out by principal investigator who is a medical doctor with PhD and has published several qualitative studies in peer reviewed Q1 journals. This will be highlighted in the COREQ & SRQR checklist. It is mentioned in the methods section that the study will be reported in accordance with these checklists. It is further highlighted in the manuscript:**

**‘The healthcare managers of the primary health centers affiliated with Primary Health Care Corporation will be contacted via email to obtain permission to approach service uses by accessing the service to participate in face-to-face interviews while they wait for their consultation in the waiting area of the health center. A separate room will be requested to be allocated by the health manager of the primary health center to conduct the interviews. The service users will be approached by the principal investigator (MA) and invited to participate in the study. When a service user agrees to participate, they will be invited to the allotted interview room. Prior to starting the interview, the purpose of the study will be explained in detail to the service user, and they will be given the opportunity to ask further questions to ensure that their participation is voluntary, informed and non-coercive. The written consent will be taken by the interviewer before starting the interview. The interviews will be recorded with permission of the service user. Once the interview has taken place all contact information about the service user will be securely destroyed and all the data collected will be anonymised. The interviews are semi-structured and will be conducted utilising an interview guide and script (supplementary document). The interviews will last between 45 to 60 minutes.**

**Analysis of the qualitative data collected from FGD and interviews:**

**The qualitative data once recorded will be transcribed verbatim and then analyzed using thematic analysis. This approach encompasses ‘interpreting, exploring, and reporting patterns and clusters of meaning within the given data’ and will be facilitated by reading and re-reading the transcripts for a full familiarization. This will be followed by application of open codes to four transcripts to identify emerging themes of relevance by two researchers (MA AND ASA). A Computer Assisted Qualitative Data Analysis (CAQDAS) package (NVivo 12 for Windows) will be utilized for this process. This will be followed by agreement by the two researchers (MA and ASA) on a set of codes which will be used with the rest of the transcripts. During this stage categories will be constructed and defined by grouping of codes. This will lead to the development of a working coding framework which will be utilized with the rest of the data and amended as necessary. The study will report the** **results in accordance with ‘Consolidation criteria for reporting qualitative research (COREQ) and ‘Standards for reporting qualitative research ‘(SRQR) guidelines.’**

**Clarity and Consistency:
The manuscript is generally clear and well-written, but minor typographical and stylistic edits are recommended. Ensure consistent terminology (for example, “service users” vs. “patients”) throughout the document for clarity.**

**Response: Thank you for the important point. The consistency has been now maintained in setting out the objectives in the introduction component as kindly suggested. The terms service users and patients had to be used interchangeably later in the text because in certain components of the protocol we had to highlight the term ‘service users’ to specifically refer to population accessing primary health care centers and registered with PHCC. And people living with chronic disease conditions had to be referred to as patients.**

**Data Availability:
The commitment to data sharing is appropriate for a protocol. Upon study completion, please ensure that all underlying data are made available in accordance with PLOS ONE’s data policy, with attention to participant privacy and ethical considerations.**

**Response:
Thank you for the important point. It is now highlighted in the ethical consideration section as kindly suggested:**

**‘Written consent will be obtained by the principal investigator (MA) prior to the start of the interviews with service users and will be recorded on the digital audio recorder with the participants’ permission. At all stages of the interview, it will be emphasised that participation is voluntary, and that the data generated will be anonymous. It will be ensured and made clear to the participants that they could withhold any information that they feel is too sensitive or withdraw from the study at any time. The interviews will be transcribed and anonymised soon after the interviews and the digital recordings of the interviews will be removed from the digital devices.** **The anonymised transcripts and digital recordings will be stored securely on a server within the clinical research department of Primary Health Care Corporation. Ethics approval was received to conduct the study from Institutional Review Board of Primary Health Care Corporation (supplementary document).**

**Additional Suggestions:**

**Consider including a brief timeline for study implementation, recruitment, and anticipated completion in the Methods section.**

**Response: Thank you for the important point. A timeline has been added in tabular form and highlighted as suggested.**
