# Supplementary material for: Exploring health literacy pertaining to general wellbeing and chronic disease management among population registered within Primary Healthcare System: A Study protocol
Source: PLoS One. 2025 Oct 7;20(10):e0333194. doi: 10.1371/journal.pone.0333194 (PMC12503302; doi:10.1371/journal.pone.0333194)
Supplement: Attachment — Submitted filename: Response to reviewer comments.docx [file pone.0333194.s004.docx]

Dr Muslim Abbas Syed

Clinical Research Department, Primary Health Care Corporation

Qatar PO Box 26555

musyed@phcc.gov.qa

Hansani Madushika Abeywickrama

Academic Editor

Plos One

Dear Hansani,

Thank you for the constructive feedback by yourself & the reviewers. We have fully considered and agree with all the reviewer comments. We have incorporated all the changes (adding additional details in methods section with references and further expanding the discussion component) which have been kindly suggested.

The comprehensive review has given us the opportunity to further strengthen the study protocol for which we are truly grateful.

The responses to individual comments are mentioned below (page 2 onwards) and changes are highlighted in the track changes of the revised manuscript.

Sincere Regards

Dr Muslim Abbas Syed

MBChB MPH MSc MS. M. ED PhD MCPS HPE FPH

**Response to individual comments**

**Reviewer 1**

**Comment: This manuscript presents a protocol for a national survey to assess health literacy in primary care in Qatar. The manuscript lacks clarity and an evidence-base in the methods, with no mention of the process of developing the data collection tools (i.e., survey and questions, plus interview guide), the process of recruitment and gaining consent from participants, and the planned and detailed data analysis (including methods of integration quantitative and qualitative datasets). In addition, there is no patient and public involvement and engagement within the protocol methods, nor is there reflection and plans for involving public and patients in the Discussion. The Methods lacks supporting references throughout, particularly in terms of survey development, and the sample size estimation is a gross estimation.**

**Response: Thank you for the important comments. We have carefully considered all the points mentioned and added the suggested details in the methods section with references and further expanded the discussion component and added additional framework (figure 2) for more clarity and detail. Your important and comprehensive feedback has helped us strengthen the protocol and add important details and relevant references supporting the evidence.**

**As kindly highlighted in your comment we have added details about the study tool (HLQ) domains and scoring, and the interview and focus group guide. While the protocol was under review, we also got the amendment approved to conduct focus group discission with health care providers (HCPs) working in PHCC. The amendment approval letter by institutional review board is also attached in the supplementary documents. This further strengthens the study as qualitative analysis (both from HCPs and service users’ perspective) will highlight important themes pertaining to the existing health literacy channels, gaps and challenges and strategies to further strengthen its implementation to promote patient centered care and further improve the quality of the services. Moreover, the sociodemographic details are also added in the HLQ. The study tools are attached as supplementary documents.**

**Furthermore, as kindly suggested references are included for both the quantitative and qualitative study tool development and interpretation (track changes). Details are added about the consent process and recruitment of the participants for interviews, FGDs and HLQ (track changes). A section on patient and public involvement is also added in the protocol. Reference for sample size is also provided.**

**Reviewer 2**

**This protocol addresses a timely and highly relevant public health concern—health literacy in chronic disease prevention and management within the primary care setting. The study’s design, ethics compliance, and clarity of objectives demonstrate strong potential to contribute valuable insights to health systems in Qatar and similar contexts. The submission is generally well-prepared for publication, pending minor clarifications.

Strengths
1. Relevance and Timeliness
The focus on health literacy in the context of chronic disease management directly aligns with national and global priorities, particularly WHO health promotion strategies.
2. Rigorous Sampling Strategy
The use of stratified random sampling from PHCC’s registry enhances representativeness across age, gender, and nationality, improving generalizability.
3. Validated Instrument
Use of the HLQ in both English and Arabic strengthens the reliability and cultural appropriateness of the tool.
4. Mixed Methods Approach
The integration of both quantitative and qualitative phases will provide a richer understanding of the issue under study.
5. Ethics and Transparency
IRB approval, consent processes, and attached documentation meet international ethical standards.
6. Document Completion
Required materials, including tools, translated forms, and supplementary documents, have been submitted.

Response: Thank you for the positive feedback and meticulous review. We are truly grateful. We have carefully considered all the important suggestions provided which has further strengthened the study protocol. Individual responses are provided below and highlighted in the track changes version of the manuscript.**

**Reviewer 2: Suggestions for Improvement
1. Missing Demographic Data in the Study Tool
The submitted Health Literacy Questionnaire (HLQ) appears to lack demographic questions (e.g., age, gender, education level, nationality). This is a critical omission for the following reasons:
• Descriptive analysis requires participant characteristics to interpret findings meaningfully.
• Subgroup analysis is necessary to identify disparities or variations in health literacy.
• Equity considerations – health literacy is often shaped by socioeconomic and cultural factors; excluding demographics limits the ability to draw meaningful conclusions and policy recommendations.
Recommendation:
Clarify whether demographic data will be obtained separately (e.g., through PHCC registry) or amend the questionnaire to include a brief demographic section. If already planned, this should be stated clearly in the methods section.**

**Response: Thank you for the important suggestion. We fully agree with you. The socio-demographic details are now added in the HLQ, and the amended version of the online tool is added as supplementary documents.**

**2. Participant Recruitment Details
• The assumed 2% response rate should be supported with rationale or precedent from similar PHCC-based studies or surveys in Qatar. This adds credibility to the feasibility of the recruitment plan.**

**Response: Thank you for the important comment. We have included reference to support the assumed 2% response rate (track changes).**

**3. Qualitative Sampling
• While non-probability sampling is acceptable in qualitative research, it would be beneficial to mention efforts to ensure diversity (gender, education, cultural background) in interview participants to reduce potential bias.**

**Response: Thank you for highlighting a very important point. We have added these details in the recruitment strategy for conducting interviews among service users and FGD with healthcare providers. While the protocol was under review, we also got the amendment approved to conduct focus group discission with health care providers (HCPs) working in PHCC. The amendment approval letter by institutional review board is also attached in the supplementary documents. This further strengthens the study as qualitative analysis (both from HCPs and service users’ perspective) will highlight important themes pertaining to the existing health literacy channels, gaps and challenges and strategies to further strengthen its implementation to promote patient centered care and further improve the quality of the services.**

**4. Data Analysis Plan
• Although HLQ scoring methods are referenced, the manuscript would benefit from a concise overview of intended statistical methods, including any subgroup analyses, correlation assessments, or use of regression models.**

**Response: Thank you for the important point. The HLQ scoring details are provided in text and tabular form now in the methods as kindly suggested.**

**5. Conceptual Framework (Figure 1)
• The figure is valuable but visually complex. A clearer flow with consistent formatting and labeled sections would improve readability for international readers unfamiliar with Qatar's healthcare system.**

**Response: Thank you for the important comment. We have added an additional section in the methods as study settings which clearly outlines the primary health care system in the state of Qatar as kindly mentioned.**

**Moreover, we have added an additional figure (figure 2) which depicts Interrelationship of health literacy levels, challenges and barriers and strategies to improve health literacy of service users that the study aims to address by triangulation of the qualitative and quantitative data collected from the study. We have added a additional section in the methods as study settings which clearly outlines the primary health care system in the state of Qatar.**

**6. Language and Style
• Minor grammatical and structural edits are recommended. For example:
o Original: “Improved compliance with treatment leading to effective management…” Suggested: “…which may improve treatment compliance and disease management.”
• Data Availability Statement: While the current version is compliant, consider stating your intention to deposit anonymized data post-study (e.g., in a public repository or institutional archive).**

**Response: Thank you for highlighting the minor grammatical and structural edits. We have thoroughly re-read the manuscript and made the necessary changes as kindly suggested. The anonymised transcripts and digital recordings will be stored securely on a server within the clinical research department of Primary Health Care Corporation. This information is now included in revised version (track changes).**

**Overall Recommendation: Minor Revisions
The study protocol is methodologically sound, ethically appropriate, and well-aligned with current health system needs. Minor adjustments related to demographic data collection, recruitment rationale, and clarity of analysis plan are needed. Once addressed, this work will make a meaningful contribution to the literature on health literacy and chronic disease management in the region.**

**Response: Thank you for the positive feedback. It is really encouraging. Your kind suggestions have strengthened the study protocol, and we are grateful.**

**Reviewer 3**

**This protocol addresses a timely and important research topic with methodological rigor. Addressing the minor clarifications below will further strengthen the manuscript and ensure its value to the scientific and public health communities.**

**Response: Thank you for the positive feedback, it is really encouraging. Your important suggestions are now incorporated in the protocol which adds relevant details and strengthened the protocol. We are truly grateful.**

**Comment: Response Rate and Potential Bias
While the protocol anticipates a very low response rate and compensates by increasing the invitation pool, it may be beneficial to elaborate further on strategies to maximize response rates (e.g., use of reminders, engagement incentives) and to discuss potential non-response bias in greater detail.**

**Response: Thank you for the important comment. We have added details (track changes) elaborating strategies to maximize the response rate as kindly suggested.** ***The identifying information (names phone numbers and HC numbers) of the targeted sample will be extracted by PHCC BHI department. The short message service (SMS) will be sent with an approved survey invitation text to the targeted sample. A reminder will be sent after a week. The invitation text message will contain a link to the online questionnaire form using Microsoft office Forms.***

**Comment: Qualitative Sampling:
The use of convenience sampling for qualitative interviews is acknowledged as a limitation. Consider discussing how heterogeneity will be maximized (e.g., purposive selection based on demographic characteristics) to enhance the credibility of qualitative findings.

Response: Thank you for the important comment. We have added these details in the recruitment strategy for conducting interviews among service users and FGD with healthcare providers. While the protocol was under review, we also got the amendment approved to conduct focus group discission with health care providers (HCPs) working in PHCC. The amendment approval letter by institutional review board is also attached in the supplementary documents. This further strengthens the study as qualitative analysis (both from HCPs and service users’ perspective) will highlight important themes pertaining to the existing health literacy channels, gaps and challenges and strategies to further strengthen its implementation to promote patient centered care and further improve the quality of the services.**

**Comment: Patient and Public Involvement:
If applicable, clarify whether patients or members of the public were involved in the development of the research questions, survey tool adaptation, or interview guide, as this is increasingly encouraged in health research.**

**Response: Thank you for the important comment. A section in the methods is added as patient and public involvement.**
