# Supplementary material for: Exploring health literacy pertaining to general wellbeing and chronic disease management among population registered within Primary Healthcare System: A Study protocol
Source: PLoS One. 2025 Oct 7;20(10):e0333194. doi: 10.1371/journal.pone.0333194 (PMC12503302; doi:10.1371/journal.pone.0333194)
Supplement: Attachment — Submitted filename: Response to reviewer comments (latest).docx [file pone.0333194.s006.docx]

Dear Editor,

We truly appreciate the invaluable feedback by yourself and the reviewers. Incorporating the kind input has strengthened the quality of the study protocol and made it more detailed and scientifically sound. All the comments have been carefully considered, and we have tried our best to address them.

Sincere Regards

Dr Muslim Abbas Syed

MBChB, MPH, MSc., MS., MCPS HPE, PhD

Response to individual comments

**Reviewer #2: The revised manuscript demonstrates significant improvement over the initial version. It now includes expanded methodological clarity, better integration of supporting literature, ethical considerations, and enhanced structure, making it a promising contribution to the field of public health in Qatar and beyond. However, several minor but important improvements are still required for optimal clarity and quality.**

**Response: Thank you for the kind comments. We truly appreciate your kind feedback which has helped us add more detailed information to improve the scientific quality of the paper.**

**Methodology Enhancements
• Ensure consistency of terminology: For example, “non-probability convenience sampling” and “purposive selection” are both mentioned—be clear on how diversity is achieved in each phase.**

**Response: Thank you for the kind comment. We have now ensured that the consistency is maintained in the manuscript. We have mentioned that the healthcare professionals and service users will be recruited from the various primary healthcare centers, ensuring that the sample is representative of the general population even though a non-probability sampling technique is utilized for the qualitative data collection.**

**• Timelines are helpful but could benefit from more detail regarding data analysis phases.
Quantitative Analysis – Clarified but Could Improve Further
• A concise overview of planned statistical analyses is still needed.
o A subgroup analyses using ANOVA or t-tests and assesses associations using regression models to evaluate factors associated with health literacy scores.
o Consider mentioning whether weighting adjustments will be used to compensate for the stratified sample.**

**Response: Thank you for the important comments regarding the quantitative analysis. We have now included additional information on descriptive and inferential data analysis as well as included template tables highlighting the various variables with P-values that will be highlighted at sub-group level analysis.**

**Qualitative Approach – Improved with FGDs
• The inclusion of themes in the topic guides is useful. Consider condensing the description of FGD logistics (e.g., room arrangements, consent) for brevity unless required by journal format.**

**Response: Thank you for your kind comment. We have included some additional logistical information as kindly suggested. Since the protocol is written for wider readership specific logistical details were avoided. But we agree with your relevant point and have included this information now.**

**• PPI is acknowledged, and a section is now added.
• However, more reflective depth is needed. Consider stating how PPI will be integrated in future research phases.**

**Response: Thank you for the kind comment. We have now included some additional information on how PPI will be integrated in future research phases.**

**Reviewer #3:**

**This revised protocol demonstrates clear improvements and addresses previous reviewer concerns effectively. The study has strong potential to contribute valuable insights to the literature on health literacy and its role in chronic disease prevention and management in primary care settings. I recommend acceptance with minor revisions, primarily to clarify the integration of mixed methods data, enhance response rate strategies, and consider final editorial polishing. I wish the authors success in conducting this important and timely study.**

**Response: Thank you for your kind words of encouragement. We are truly grateful for your feedback, which has been constructive, accurate and strengthens the quality of the study protocol. We have carefully considered all your comments and tried our best to address them.**

**Minor Clarifications and Recommendations
Data Integration Approach:
While the manuscript mentions the triangulation of quantitative and qualitative findings, it would benefit from further elaboration on how this integration will be operationalized (e.g., through matrix comparison, joint displays, or narrative synthesis).**

**Response: Thank you for this important point. We have added details about narrative synthesis and have also described how the evidence based Socioecological Model (SEM) can be potentially utilized to interpret the qualitative findings (the factors affecting the health literacy of service users accessing primary healthcare services) at intra-personal, inter-personal, institutional, community and policy level. The findings of the study will populate the framework. The findings of the HLQ (survey) provide invaluable information at subgroup population level which feeds into the intrapersonal factors affecting the health literacy of service users accessing the primary health care services and can be also presented in detail as an individual publication resulting from this study project. Considering your kind suggestion we have included an additional brief paragraph explaining the triangulation of qualitative and quantitative data.**

**Qualitative Sampling and Saturation:
The authors indicate that interviews will continue until data saturation is reached. To strengthen this, it is recommended to briefly describe how saturation will be monitored or defined during data collection.**

**Response: Thank you for highlighting a very important point. It is now described that saturation is achieved in qualitative data collection when no new additional information is provided by the new participants (service users) or no new ideas and themes are discussed. A point where the same concepts are being discussed. This concept is established and well documented in qualitative research and now a reference is also provided.**
 **Strategies to Improve Response Rates:
Given the very low expected response rate (2%), it may be useful to discuss any additional measures to enhance participation, such as incentives, follow-up reminders, or community engagement strategies.**

**Response: Thank you for the important point. The strategies to enhance participation are now expanded and further described as kindly suggested.**

**Conceptual Frameworks (Figures 1 & 2):
The addition of Figure 2 is appreciated; however, the visual complexity of the frameworks may be a challenge for general readers. Simplifying the layout and labeling the flow components more clearly could improve accessibility.**

**Response: Thank you for the important comment. We also included a simplified figure 1 and interlinked it with figure 2 for clarity. The conceptual framework was included as they will form the basis of reporting the key findings of the study for publication.**

**Data Availability Statement:
The current statement indicates that data will be made available upon study completion. The authors may consider specifying the repository (e.g., institutional archive or open-access platform) where de-identified datasets will be deposited, in line with open science principles.**

**Response: Thank you for the important point. We have now included this information.**

**Language and Style:
While the manuscript is generally well-written, a final proofreading may help correct minor typographical inconsistencies and improve flow, particularly in longer paragraphs in the introduction and discussion sections.**

**Response: Thank you for the important point. As kindly suggested, a final proof reading was done. Thank you for this important suggestion.**
