# Supplementary material for: Exploring health literacy pertaining to general wellbeing and chronic disease management among population registered within Primary Healthcare System: A Study protocol
Source: PLoS One. 2025 Oct 7;20(10):e0333194. doi: 10.1371/journal.pone.0333194 (PMC12503302; doi:10.1371/journal.pone.0333194)
Supplement: Attachment — Submitted filename: Response to reviewer comments (4th revision).docx [file pone.0333194.s008.docx]

**Response to reviewer comments**

**Reviewer #3: Thank you for the opportunity to review this well-constructed and timely study protocol addressing health literacy in primary healthcare settings in Qatar. The manuscript presents a clear rationale, an appropriate mixed-methods approach, and demonstrates careful consideration of both methodological rigor and ethical compliance. The following comments summarize the strengths of the protocol and provide suggestions for improvement to enhance clarity, replicability, and alignment with PLOS ONE’s expectations.**

**Response: Thank you for the detailed review and useful suggestions in the previous reviews. We are grateful for your time and expert input. Your feedback has made the protocol very much detailed and scientifically robust. We have also considered your minor revision suggestions in this 4^th^ round of review and have incorporated all the changes kindly suggested by you.**

**1. Rationale and Research Questions

The manuscript presents a strong rationale for the study, supported by a comprehensive review of the literature. The authors effectively highlight the significance of health literacy in the context of chronic disease management and general wellbeing, particularly in multicultural populations. The research questions and objectives are clearly defined, relevant, and appropriately justified. The use of the Socioecological Model (SEM) as a conceptual framework adds depth and relevance to the study design.**

**Response: Thank you for the kind comments. Your suggestions in the previous reviews were very helpful and strengthened the protocol rationale and highlighted the relevant frameworks (adding additional details). We are grateful.**

**2. Methodological Soundness and Analytical Strategy

The mixed-methods approach—integrating the Health Literacy Questionnaire (HLQ) and qualitative interviews/focus groups—is well-justified and methodologically sound. The sampling strategy for the quantitative component is detailed and appropriate, with clear justification for oversampling due to the anticipated low response rate. The inclusion of subgroup weighting and sensitivity analysis is commendable and strengthens the analytical plan.**

**Response: Thank you for the kind comments. The inclusion of subgroup weighting and sensitivity analysis was one of your kind suggestions in the 3^rd^ round of review previously. Thank you for your feedback which has strengthened the protocol scientifically.**

**The qualitative components are described in detail, including participant recruitment, data collection procedures, thematic analysis approach using NVivo, and adherence to COREQ and SRQR guidelines. The triangulation of quantitative and qualitative findings is a major strength and is well-explained.**

**Response: Thank you for the kind comments and encouragement.**

**However, I recommend the following minor improvements:

Statistical power calculation: Although the sample size is large, inclusion of a formal power analysis for the HLQ component would further validate the sampling rationale.**

**Response: Thank you for the important comment. We have added the following details as kindly suggested in the sampling strategy section:**

**‘****A sample size of only 2000 will detect a very small difference of 0.1 in the mean score of a specific domain (ranging between 1 and 4 with a standard deviation of 1) between two groups with an estimated Beta power of 0.99 at alpha of 0.05. Therefore, the targeted sample size of 6000 will detect virtually any difference or effect’.**

Clarify exploratory elements: Consider clearly identifying which components of the analysis (particularly within the qualitative arm) are exploratory, and how these will be distinguished in reporting.

**Response: Thank you for the comment. We have provided more details as kindly highlighted in your important point in the interpretation of qualitative findings (the FGD and interviews will feed the SEM model). It is now highlighted in that section as outlined below:**

**‘The qualitative analysis will be interpreted utilizing the Socioecological Model (SEM) which will provide to explore the wider determinants of health literacy of service users accessing the primary health care services. SEM is a widely accepted framework for comprehending and describing health determinants and in its entirety can be effectively utilized to explore the factors associated with health literacy of service users nested within the SEM. Although the SEM is widely acknowledged as a framework for understanding wider health determinants, there are only a few examples embedding health literacy in socioecological context. The existing literature only partially explores determinants nested within the SEM and does not investigate the model’s entirety when examining factors related to health literacy of service users accessing primary care services.** **The state of Qatar has a highly developed and extensive primary care health system, Primary Health Care Corporation (PHCC) which is composed of 32 primary health care centers which are scattered throughout the country. PHCC has the prime objective to deliver universal health coverage to its registered population. Each primary health care center is equipped with modern healthcare facilities managed by an international multidisciplinary team of health care professionals delivering the highest standards of primary health care services to a diverse multi-ethnic population (comprising of approximately 88% expats). However, studies report that despite the strong primary health care infrastructure, Qatar has a high prevalence of non-communicable disease and challenges associated with uptake of the services among the population registered with PHCC with diverse ethnic backgrounds. The model will be populated by the important themes emerging from the qualitative interviews with the patients and FGDs with HCWs to highlight the intrapersonal, interpersonal, Institutional, Community and Policy level factors as illustrated in figure 1.**

**3. Feasibility and Replicability

The study appears feasible within the stated timeline and is supported by access to a large, registered population within PHCC. Methodological descriptions, including sampling, recruitment, data management, and ethical procedures, are sufficiently detailed to allow for replication. The use of a validated instrument (HLQ) and the structured thematic analysis framework provide a solid foundation for reproducibility.**

**Response: Thank you for the kind comments. The previous reviews and kind suggestions have substantially strengthened the protocol.**

**Suggestions:**

**A brief mention of interviewer training (e.g., for conducting interviews and FGDs) would enhance transparency.**

**Response: Thank you for highlighting an important point. The interviews and FGD will be conducted by Principal investigator who is a medical doctor with multiple master’s degrees and PhD. He is a published researcher and has substantial experience of publishing qualitative research in Q1 high impact journals. This is also a criterion in the COREQ and SRQR checklist. We have added this information as highlighted in your kind comment.**

**‘The interviews will be conducted by PI. He has the relevant academic qualifications and substantial experience of conducting qualitative research and publishing in high impact journals. This is in accordance with the criterion of COREQ and SRQR regarding standards required by the researcher conducting qualitative research.’

Consider including a brief outline of data monitoring or audit mechanisms, if any, to strengthen quality assurance.**

**Response: Thank you for highlighting a very important point. We have added a paragraph on quality control and good practice measures:**

**‘To ensure the ethical and regulatory integrity of the study, oversight mechanisms will be implemented throughout its duration. A designated research monitor from the Clinical Research Department will conduct periodic monitoring and auditing activities. These will verify that the study is being conducted in accordance with the protocol approved by the Institutional Review Board (IRB), and in compliance with Good Clinical Practice (GCP) guidelines and Policies, Regulations and Guidelines for Research Involving Human as outlined by Ministry of Public Health (MoPH) Qatar. Monitoring will include the identification and documentation of any protocol deviations or instances of non-compliance. All findings will be addressed promptly to uphold participant safety and maintain the scientific validity of the research.’**

**4. Data Availability

The authors appropriately address data availability in accordance with PLOS ONE’s policy. While no datasets are yet generated, the authors commit to making de-identified data available upon study completion, with proper ethical safeguards. This is acceptable at the protocol stage.**

**Response: Thank you for the kind comments.**

**5. Language and Presentation

The manuscript is generally well-written and intelligible. The use of standard academic English is appropriate, and the document is logically organized. Minor typographical and stylistic issues exist (e.g., occasional inconsistent use of “patients” vs. “service users”), but these do not impede understanding. A final proofreading prior to acceptance is recommended to correct minor inconsistencies.**

**Response: Thank you for the kind comments. A final proof reading is now done.**

**6. Ethical Considerations

The study demonstrates full compliance with ethical standards. IRB approval is clearly stated, informed consent procedures are appropriate, and participant confidentiality is well-addressed. The manuscript includes clear information about how data will be anonymized, stored, and handled securely.**

**Response: Thank you for the kind comments. Your input was indeed very useful and has further strengthened the protocol.**

**7. Additional Suggestions (Optional)

The inclusion of figures and conceptual models (SEM and health literacy framework) adds value. Ensure these are high-resolution and clearly labeled in the final submission.

Consider adding a brief dissemination plan, outlining how findings will be shared with stakeholders (e.g., healthcare providers, policymakers, or the public) to maximize impact.**

**Response: the following section is added as dissemination plan as kindly suggested:**

**Dissemination Plan**

**Upon completion of the study, findings will be disseminated through multiple channels to ensure a broad and meaningful impact. Results will be submitted for publication in peer-reviewed journals and presented at relevant scientific conferences. In addition, tailored summaries will be shared with key stakeholders, including healthcare providers and policymakers, to inform practice and decision-making. Where appropriate, public-facing materials will be developed to communicate findings to the public, thereby enhancing transparency and promoting community engagement with the research outcomes.**
